# Modular reorganization of the global network of gene regulatory interactions during perinatal human brain development

**DOI:** 10.1186/s12861-016-0111-3

**Published:** 2016-05-12

**Authors:** Jimena Monzón-Sandoval, Atahualpa Castillo-Morales, Araxi O. Urrutia, Humberto Gutierrez

**Affiliations:** School of Life Sciences, University of Lincoln, Lincoln, LN6 7DL UK; Department of Biology and Biochemistry, University of Bath, Bath, BA2 7AY UK; Milner Centre for Evolution, University of Bath, Bath, BA2 7AY UK

**Keywords:** Gene coexpression network, Brain transcriptome, Human brain development, Gene regulation

## Abstract

**Background:**

During early development of the nervous system, gene expression patterns are known to vary widely depending on the specific developmental trajectories of different structures. Observable changes in gene expression profiles throughout development are determined by an underlying network of precise regulatory interactions between individual genes. Elucidating the organizing principles that shape this gene regulatory network is one of the central goals of developmental biology. Whether the developmental programme is the result of a dynamic driven by a fixed architecture of regulatory interactions, or alternatively, the result of waves of regulatory reorganization is not known.

**Results:**

Here we contrast these two alternative models by examining existing expression data derived from the developing human brain in prenatal and postnatal stages. We reveal a sharp change in gene expression profiles at birth across brain areas. This sharp division between foetal and postnatal profiles is not the result of pronounced changes in level of expression of existing gene networks. Instead we demonstrate that the perinatal transition is marked by the widespread regulatory rearrangement within and across existing gene clusters, leading to the emergence of new functional groups. This rearrangement is itself organized into discrete blocks of genes, each targeted by a distinct set of transcriptional regulators and associated to specific biological functions.

**Conclusions:**

Our results provide evidence of an acute modular reorganization of the regulatory architecture of the brain transcriptome occurring at birth, reflecting the reassembly of new functional associations required for the normal transition from prenatal to postnatal brain development.

**Electronic supplementary material:**

The online version of this article (doi:10.1186/s12861-016-0111-3) contains supplementary material, which is available to authorized users.

## Background

Development of the human nervous system is a complex and precisely regulated process that occurs over a prolonged period of time and depends on a strict temporal and regional coordination of complex patterns of gene expression. As the developmental programme unfolds, genes modify their level of expression in the brain at specific times in response to changing demands for a variety of cellular functions. Up to 89.9% of brain expressed genes have been shown to be temporally differentially expressed between any two periods across regions, with 85.3% of genes being differentially expressed at any two different time points across areas in the neocortex alone [1, 2]. Waves of intense variation in gene expression are particularly pronounced in specific stages of development. In the rat brain model, for instance, for most genes, the most dramatic changes in level of expression occur early in postnatal life (1–2 weeks) and plateau thereafter [1].

Observable changes in gene expression profiles throughout development are determined by an underlying network of precise regulatory interactions between individual genes [3]. Elucidating the organizing principles that shape the whole network of gene regulatory interactions that ultimately instruct organismal development is one of the central goals of developmental biology. In this context, it is of critical important to ascertain whether the gene regulatory architecture driving development is itself a constant or variable feature of the developmental programme. Most cellular processes are the result of a complex assembly of molecular and genetic components acting in concert [4] suggesting the need of stable regulatory interactions between defined groups of genes throughout development. On the other hand, many genes have the potential to participate in multiple separate and sometimes seemingly unrelated biological functions [5], also suggesting the existence of occasional events of regulatory reassembly giving rise to the emergence of new functional associations.

Here we ask whether global changes in gene expression profiles during development are primarily the result of a dynamic driven by a fixed regulatory architecture, or alternatively, the result of temporally defined waves of regulatory reorganization.

Genes linked by regulatory interactions tend to display similar expression patterns reflecting their functional association [6, 7]. This coordinated expression can be readily detected by looking at existing correlations in expression levels between groups of genes across a series of suitable chosen tissue samples. Along these lines, clustering analysis based on co-expression patterns has been used to identify groups or modules of correlated genes that may form molecular complexes, pathways, or participate in common regulatory and signalling circuits [8–14]. Apart from revealing functional interactions among groups of genes, gene co-expression also provides information on the underlying regulatory architecture associated to a global expression profile as co-expressed genes are likely to be under the concerted control of a common complement of transcriptional regulators [15–17].

In agreement with studies in other cellular systems, during development, the human brain transcriptome has also been shown to be organized into distinct coexpression networks of functionally related genes [10, 18]. These networks are generally assumed to behave as single expression units where co-regulated genes vary jointly in their level of expression across development in response to changing demands of their collective functions [18]. Current studies tend to suggest that the stability of the coexpression structure of the transcriptome is an essential condition for the normal function of cells and tissues as changes in the correlated status of groups of genes have been linked to a range of diseases and pathological conditions including cancer, obesity, degenerative conditions and neuropsychiatric disorders as well as progressive genome instability associated to age-related functional decline [11, 12, 14, 19–25].

In this study, we analyse genome wide expression data derived from the developing human brain cortex at several stages across eight cortical regions and examine the relationship between changes in gene expression profiles and the correlation structure of the developmental transcriptome. Clustering analysis of expression profiles show that gene expression throughout development is divided into two clearly defined temporal domains before and after birth. By comparing the coexpression structure of all cortical regions in the perinatal transition, we show that this sharp division between foetal and postnatal profiles is not the result of pronounced changes in the level of expression of existing networks of co-regulated genes. Instead we demonstrate that the perinatal transition is marked by the widespread regulatory rearrangement within and across existing gene clusters giving rise to the emergence of new functional groups. Our results reveal an acute regulatory reorganization of the brain transcriptome occurring specifically at birth and reflecting the reassembly of new functional associations potentially required during the transition from prenatal to postnatal brain development.

## Results

**Fig 1:**
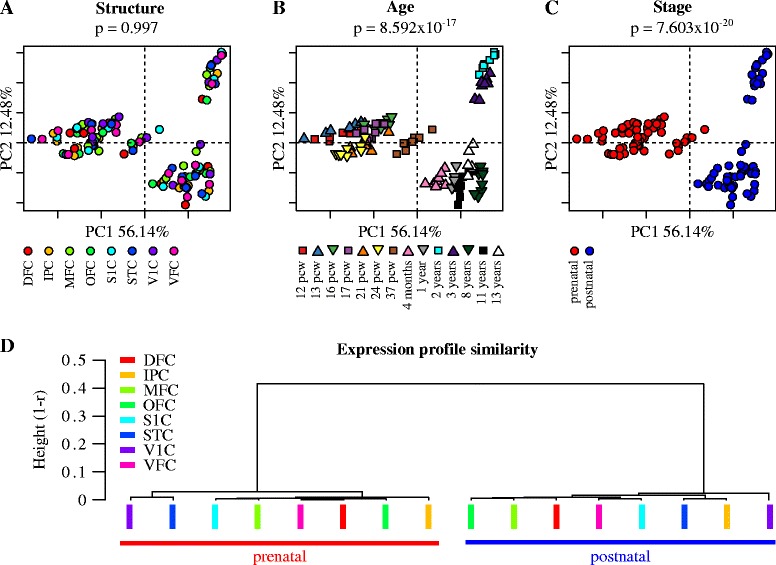
Developmental stage, but not anatomical structure contributes to the greatest component of variance in gene expression profiles. Principal component analysis splitting samples of expression data by either **a** structure, **b** post conception age or **c** prenatal/postnatal stage. Each plot shows the first and second components (together contributing to 68.83% of variance). Kruskal-Wallis test was carried out on PC1 to test for associations between this component and either, structure, post conception age or prenatal/postnatal stage. Associated p values are indicated. **d** Relatedness between average postnatal or prenatal expression profiles across anatomical regions. Average expression per gene per cortical region was obtained for either prenatal or postnatal samples across all analysed cortical regions. Unsupervised hierarchical clustering was conducted using pairwise correlations between all resulting average expression profiles as a measure of similarity. Note, that the average expression profiles of any two prenatal regions are mores similar to each other, than they are to themselves across the perinatal boundary. Acronyms for brain structures: Dorsolateral prefrontal cortex (DFC), Posteroinferior parietal cortex (IPC), Medial prefrontal cortex (MFC), Orbital frontal cortex (OFC), Primary somatosensory cortex (S1C), Posterior superior temporal cortex (STC), Primary visual cortex (V1C) and Ventrolateral prefrontal cortex (VFC)

We examined RNA seq expression data obtained from the NIMH Transcriptional Atlas of Human Brain Development (http://www.brainspan.org/). We selected 112 samples corresponding to eight brain structures for which there was available data across 14 pre and postnatal developmental stages (post-conception weeks 12, 13, 16, 17, 21, 24 and 37; 4 months after birth as well as 1, 2, 3, 8,11 and 13 years of age). In order to maximize the number of genes included in this study, all genes displaying zero variance across samples were removed from the analysis, resulting in a total of 18526 genes. We started by asking whether changes in gene expression profiles during brain cortex development are mostly associated to regional or temporal differences. To this end we carried out a principal component analysis splitting samples by either region or post conception age. Using the first and second components (together contributing to 68.83% of variance) we found no significant association between variations in gene expression profiles and anatomical structure (Kruskal Wallis test, *p* = 0.997, Fig. 1a). By contrast, the global expression pattern showed a highly significant association with post conception age (Kruskal Wallis test, *p* = 8.592 ×10^-17^, Fig. 1b) demonstrating a more prominent contribution of the developmental stage to the observed changes in gene expression than differences attributed to regional variations. Furthermore, when we split expression data into prenatal and postnatal samples, the association between expression profiles and these two developmental windows was even more pronounced (Kruskal Wallis test, *p* = 7.603 × 10^-20^, Fig. 1c).

These results show that the single greatest component of gene expression profile variance corresponds to the developmental stage of the brain rather than anatomical structure. More specifically, these results reveal a distinctly pronounced transcriptional profile shift between prenatal and postnatal expression irrespective of brain region.

To directly test the apparent partition of expression profiles between prenatal and postnatal stages, we assessed the transcriptional relatedness between all brain regions, averaging, for each brain region, prenatal and postnatal expression per gene, resulting in a total of 16 average expression profiles; one for each of the eight brain regions at either prenatal or postnatal stages. Using these profiles, we calculated correlation matrices of pairwise comparisons followed by unsupervised hierarchical clustering. This analysis revealed two highly correlated expression profiles sharply dividing foetal and postnatal stages (Fig. 1d and Additional files 1 and 2). These results show that any two brain regions are more similar to each other within each developmental window than they are to themselves across the perinatal boundary and demonstrate the existence of two distinct global expression patterns characterizing the prenatal and postnatal development in nervous tissues irrespective of which anatomical region they belong to.

**Fig 2:**
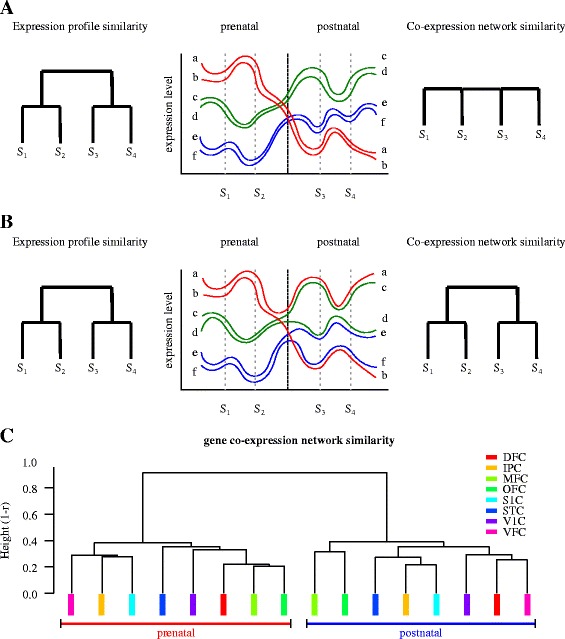
Alternative models explaining the observed switch in the global expression profile sharply dividing prenatal and postnatal brain development. Patterns of expression of six hipothetical genes in two prenatal (S1, S2) and two postnatal (S3, S4) samples. **a** Switch in the global expression profile under constant regulatory interactions resulting from a pronounced change, during the perinatal boundary, in the overall level of expression of existing, but otherwise cohesive gene clusters. **b** A similar switch in the global expression profile resulting from a widespread remodelling of the underlying regulatory structure leading to the reassembly of new functional clusters. **c** Relatedness between actual coexpression profiles across anatomical regions. Gene coexpression matrices per cortical region were obtained for both prenatal and postnatal developmental windows. Unsupervised hierarchical clustering was conducted using pairwise correlations between all resulting coexpression matrices as a measure of similarity. Note that the coexpression structure of any two prenatal (or postnatal) regions are mores similar to each other, than they are to themselves across the perinatal boundary

The observed switch in the global expression profile sharply dividing the prenatal and postnatal developing human nervous system can be alternatively explained as the result of two underlying processes: A) a pronounced change, during the perinatal boundary, in the overall expression profile driven by an otherwise constant network of regulatory interaction between genes (regulatory static model, Fig. 1a) or B) a widespread reorganisation of the regulatory programme leading to the overall reassembly of gene regulatory interactions (regulatory reorganisation model, Fig. 2b).

We can discriminate between these two models, by using the overall gene coexpression structure of the transcriptome at a given developmental window as a measure of the underlying gene regulatory architecture, and use this coexpression structure to compare between prenatal and postnatal stages. Under the static network of regulatory interactions model, we would expect the pattern of coexpression for individual brain regions to remain similar at both prenatal and postnatal stages (Fig. 2a). On the other hand, under the assumption of a regulatory reorganization occurring at birth, we would expect the split in expression profiles between prenatal and postnatal stages to be accompanied by a concurrent split in the coexpression structure precisely at the perinatal boundary (Fig. 2b).

To this end, we used a weighed a gene coexpression network analysis approach (WGCNA), where the coexpression structure of the transcriptome of a given brain region can be represented as the Pearson correlation matrix of all possible pairs of genes across a number of developmental time points [8–13]. Accordingly, we obtained the coexpression matrices of each brain region for prenatal and postnatal stages separately, resulting in a total of 16 different coexpression matrices: one for each of the eight brain regions at either prenatal or postnatal stages. The resulting coexpression matrices where then used to conduct an average linkage hierarchical clustering analysis and defined similarity between any two coexpression matrices as (1-R), where R is again the Pearson coefficient derived from correlating any two coexpression matrices.

As shown in Fig. 2c and Additional file 3: Figure S3, clustering analysis of brain regional samples based on their coexpression architecture shows a clear split between pre and postnatal samples, with coexpression structures within each developmental stage resembling more each other irrespective of brain region, than the same region resembling itself across these two developmental windows (see Additional file 3: Figure S3 for the corresponding correlation matrix). This result demonstrates an overall reorganization of the coexpression structure of the brain transcriptome, as the developmental program crosses the perinatal boundary, further revealing a widespread remodelling in the gene regulatory structure of the developmental programme between late prenatal and early postnatal stages.

**Fig 3:**
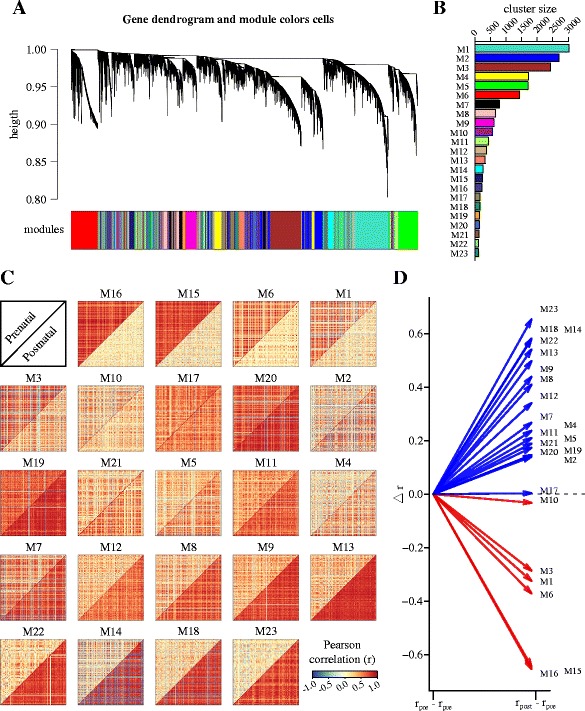
Differential coexpression network dendrogram and gene modules. **a** Dendrogram showing average hierarchical clustering based on the topological overlap of the adjacency matrix based on coexpression differences (see methods) and the corresponding gene modules indicated in different colours underneath. **b** Histogram showing cluster size (number of genes) per module. **c** Heat maps of the Pearson correlation coefficients between all possible gene pairs contained within each module. Each heat map shows prenatal and postnatal coexpression separately (upper and lower diagonal respectively). Colour scale for correlation coefficients is shown at the top left corner of this panel. Modules are arranged from left to right and top to bottom according to the median correlation difference between prenatal and postnatal stage. **d** Graph showing the change in the average correlation of each module between pre and postnatal stages. Each arrow represents the difference in postnatal average correlation relative to the prenatal stage. 17 out of 23 differentially coexpressed modules displayed increased correlated expression in the postnatal stage with six modules showing reduced correlated activity in transition from pre to postnatal development

In order to characterize the pattern of regulatory changes occurring during the perinatal boundary, we conducted differential coexpression analysis as described by Tesson et al, [26]. This method groups genes together when their correlations with the same sets of genes change between different conditions. Briefly, we obtained the overall coexpression matrices for either prenatal or postnatal stages, each one comprising data from all seven ages and eight brain regions and obtained a matrix of adjacency difference (D) as defined by Tesson et al (absolute difference of the signed squared correlation between conditions). A topological overlap matrix based on the differential coexpression matrix was then calculated followed by hierarchical clustering to identify modules of differentially coexpressed genes (Fig. 3a). This analysis identified a total of 27 modules of differential coexpression that were further merged if their eigengene correlations were high (R > 0.9, see methods) resulting in a total of 23 differential coexpression modules ranging in size from 115 to 3021 genes (Fig. 3a and b). A close inspection of the correlation heat-maps of the resulting clusters confirms pronounced changes in the correlated structure of each module in the transition between prenatal to postnatal development with most modules displaying an overall increase in correlated activity in the postnatal stage (Fig. 3c). We quantified this effect by simply measuring the change in the average correlation of each module between pre and postnatal stages (Fig. 3d) and found that 17 out of 23 differentially coexpressed modules displayed a significant increase in correlated expression in the postnatal stage with 6 modules showing reduced correlated activity in the same developmental stage relative to prenatal development. To determine the statistical significance of the observed correlation changes in all modules, we performed a permutation analysis as described and implemented by Tesson et al. 2010 where 1000 permutations are carried out on the expression values of each module and the proportion of changes in correlation higher than the one observed is determined [26] This analysis revealed that the observed changes were significant at this level (p < 0.001) for all 23 modules.

Together, these results demonstrate an overall reassembly of the regulatory structure of the brain transcriptome in the perinatal boundary, and show that this reassembly is itself organized into discrete modules or clusters of genes undergoing intensive regulatory reorganization.

**Fig 4:**
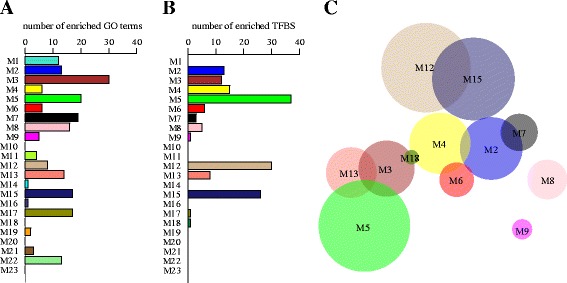
Regulatory reorganization modules are enriched in defined sets of biological processes and targeted by specific sets of transcription factors. **a** Bar plot showing the number of significantly enriched GO terms per individual module. Statistical significance in enrichment of surveyed biological process GO terms (*n* = 106) was determined by means of a Z-score test, where the expected number of genes annotated to individual GO terms was determined numerically by comparing with 10 000 equally sized random samples of genes. Significance threshold was adjusted for multiple testing by Benjamini Hochberg correction (FDR < 0.05). **b** Bar plot showing the number of significantly enriched of transcription factor target motifs per individual reorganization module using Webgestalt’s transcription factor target (TF) enrichment toolkit. **c** Venn-Euler diagram showing the relative number of TF binding motifs statistically overrepresented within each individual module. The area of each circle represents the number of enriched TF binding sites in each module and the overlap represents the relative proportion of overlapping motifs between modules. Note that each module targets a quasi-exclusive set of biological functions with rare overlap between modules

In order to test the functional significance of the observed modular reorganization of the brain transcriptome in the perinatal boundary, we asked whether each regulatory reorganization module targeted a defined set of biological functions. To this end, we determined the number of gene ontology (GO) terms, within the biological process category, statistically overrepresented within each module (see methods). As shown in Fig. 4a, 19 out of 23 regulatory reorganization modules displayed significant enrichments in one or more specific biological processes (see Additional files 4: Table S1 and 5: Table S2). This result shows that the observed regulatory reorganization of the transcriptome in the perinatal boundary is organized into discrete regulatory remodelling networks, each significantly enriched in defined sets of biological functions.

Potential molecular drivers of this global regulatory remodelling during the perinatal transition could in principle include changes in expression of transcription factors specifically targeting individual reorganization modules. To test this hypothesis we looked at enrichment of transcription factor (TF) targets among all detected reorganization modules. As shown in Fig. 4 b, using the transcription factor target enrichment toolkit of Webgestalt [27] we found 13 modules significantly enriched in defined sets of TF binding sites. Fig. 4c shows a Venn-Euler plot where the area of each circle represents the number of enriched TF binding sites in each module and the overlap represents the relative proportion of overlapping binding sites between modules. As can be seen in the graph, this analysis reveals that each module is enriched an almost exclusive set of transcription factor binding sites with rare overlaps between modules. This finding strongly suggests that each module is under the transcriptional regulation of a quasi-exclusive set of transcription factors. Indeed, a closer inspection of this analysis reveals 135 enriched TF binding motifs in total across all modules. Of these, 116 were each exclusively enriched in single modules with no overlap with any other module, (see also Additional file 6: Table S3). In order to assess the statistical significance of this strong bias towards non-overlapping transcription factor binding motifs, we performed 10 000 permutations of the distribution of enriched TF bindings sites across modules and found that the observed number of module-specific (non-overlapping) transcription factors was 11 times higher than expected purely by chance (expected = 10.35, Std dev=5.63, *p*<< 0.0001). These results demonstrates that the regulatory reorganization modules identified are each targeted by a quasi-exclusive, non-overlapping, set of transcriptions factors and suggest that the observed changes in the pattern of coordinated activity in these modules is brought about by the engagement (or disengagement) of distinct sets of module-specific transcriptional regulators.

**Fig 5:**
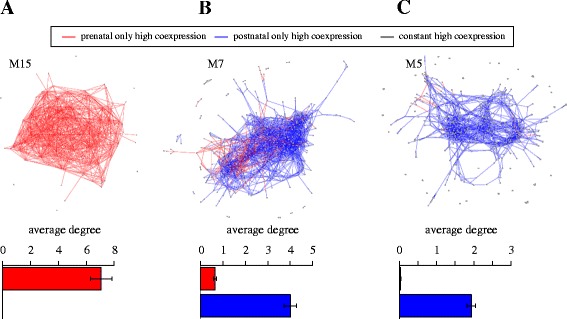
Examples of network structure rearrangements during the perinatal transition. Illustrative examples of the internal structure of the regulatory reorganization in three representative modules. Each module or gene cluster is represented as a graph of coexpression relationships, where nodes represent genes and edges represent high correlations (*R* > 0.95) between pairs of genes. Red edges represent prenatal-only high correlations, blue edges represent postnatal-only high correlations and black edges represent constant (prenatal and postnatal) high correlations. **a** Module M15 shows an almost exclusive transition from high to low correlation in all involved genes in the transition from prenatal to postnatal development. By contrast module M7 **b** shows a transition from high to low coordination between genes in a subnetwork with a simultaneous transition from low to high correlated activity in a second sub-network. Module M5 **c** shows an almost exclusive transition from low to high correlation in all genes involved during the transition from prenatal to postnatal brain development. Inset: bar plots showing the mean number of prenatal (red) or postnatal (blue) edges per node (degree) in each module

Within each module, the observed regulatory reorganization involved either events of increased or decreased coordinated activity between individual gene pairs or a combination of both. We can illustrate this in the examples shown in Fig. 5 corresponding to modules M15, M7and M5, where each module is represented as a graph, with nodes representing genes and edges represent an existing high correlation (*R* > 0.95) between the indicated pair of genes. Red edges represent prenatal-only high correlations, whereas blue edges represent postnatal-only high correlations. Module M15 shows an overall transition from high to low correlated activity for all involved gene pairs in the transition from prenatal to postnatal development. By contrast module M7 shows a transition from high to low coordination between genes in a subnetwork accompanied by a transition from low to high correlated activity in a second sub-network. Module M5 on the other hand shows an almost exclusive perinatal transition from low to high correlation in all genes involved.

Taken togeher our results demonstrate an acute and modular regulatory reorganization of the brain transcriptome occurring at birth and reflecting the reassembly of new functional associations potentially required during the transition from prenatal to postnatal brain development.

## Discussion

The development of the nervous system is a highly complex process, involving the coordinated regulation of thousands of genes instructing the generation, migration, final location, and connectivity of neurons and circuits. As the developmental program unfolds, gene expression patterns vary widely depending on the specific developmental trajectories of different neural structures [1, 2]. Global changes in gene expression profiles throughout development are strictly determined by an underlying network of precise regulatory interactions between individual genes and understanding the organizing principles that define this regulatory network is one of the central goals of developmental biology.

In this study we specifically asked whether the regulatory architecture driving global changes in expression profile during nervous system development is a constant or variable feature of the developmental programme. To address this problem we focused on the coexpression structure of the transcriptome as a measure of its regulatory architecture at defined stages during development. To this end, we used existing expression data derived from the developing human brain cortex in prenatal and postnatal stages and examine the relationship between changes in gene expression profiles and the underlying correlation structure of the developmental transcriptome.

We started by examining existing variations in global expression profiles across different developmental stages and cortical areas. Our results reveal a sharp split occurring at birth across brain areas showing two distinct families of expression patterns charactering prenatal and postnatal development respectively. This result is consistent with previous findings in the rat model where gene expression profiles throughout development are also divided into two clearly defined temporal domains. In the rat model however, this split in expression profiles occurred between one and weeks after birth [1].

This shift in the overall expression pattern is likely to reflect a major transition in the developmental trajectory and could be explained as resulting from two alternative underlying processes: A) a marked change, during the perinatal boundary, in the overall expression profile driven by an otherwise fixed network of regulatory interaction between genes or B) a widespread reorganisation of the regulatory programme, leading to the overall reassembly of gene regulatory interactions and the emergence of new functional associations.

To discriminate between these two models we compared the coexpression structure of the transcriptome between prenatal and postnatal stages for all cortical regions. Under the constant network of regulatory interactions model we would predict little differences in the coexpression structure across regions and between prenatal and postnatal development. Contrary to this prediction, we found a clear split in the correlation structures sharply dividing prenatal and postnatal samples precisely coinciding with the previously oberved divide between prenatal and postnatal expression profiles. This result is consistent with the regulatory reorganization model where the developmental gene expression programme undergoes an overall reassembly of gene regulatory interactions.

Gene coexpression analysis has been widely used to gain insights into the functional organization of transcriptomes across tissues, conditions and species [8–14], and studies of the brain developmental transcriptome have revealed distinct coexpression networks displaying clearly defined patterns of temporal expression [18]. These networks are widely assumed to behave as single expression units composed of cohesive groups of co-regulated genes [18] and expected to represent a constant feature of the normal developmental programme. In line with this notion, changes in the correlated status of groups or networks of genes have been linked to regulatory dysfunctions associated to the onset or progression of various disease states and pathological conditions such as cancer, obesity, neurodegeneration and neuropsychiatric disorders as well as potential genome instability associated to age-related functional decline [11, 12, 14, 19–25]. This link between pathological dysfunctions and changes in the global network of regulatory interactions suggest that the stability of the coexpression structure of the transcriptome is an essential condition for the normal function of cells and tissues. In this study, we found an overall reorganization of the coexpression structure of the brain developmental transcriptome, not as part of a pathological process but as part of the normal developmental trajectory of the nervous system.

Using differential coexpression analysis, we further characterized the overall remodelling of the regulatory structure of the brain transcriptome at birth and found that this reassembly is itself structured into discrete modules or clusters of genes undergoing intensive regulatory reorganization.

In order to gain insights into the functional coherence of the observed modular reorganization of the brain transcriptome at birth, we asked whether these reorganization clusters targeted specific biological functions. Gene ontology enrichment analysis revealed that each module targets a separate set of biological functions, with little functional overlap between modules. This result shows that the observed regulatory reorganization of the transcriptome in the perinatal boundary is organized into discrete clusters each involved in the regulatory remodelling of defined sets of biological functions.

Our finding that this transition is organized into discrete modules targeting distinct sets of biological functions strongly suggests the emergence of new functional associations required for the normal transition from prenatal to postnatal brain development. Thus for instance module M15 displays an overall reduction of correlated activity between its genes in the transition from prenatal to postnatal development. Interestingly, this module shows a distinct, and statistically significant, overrepresentation of genes involved in cell cycle, mitosis and cell proliferation functions. This reduction in the level of coordination between genes directly involved in proliferative functions could potentially reflect corresponding differences in the level of engagement of proliferative activity during prenatal and postnatal nervous system development. Indeed, cell proliferation is particularly pronounced during embryonic and late foetal stages of nervous system development as neuronal progenitor cells proliferate and their populations expand to eventually differentiate into mature post-mitotic neurons. At postnatal stages, proliferative activity virtually ceases for neural precursors and remains restricted to the sustained but low production of both astrocytes and oligodendrocytes [28, 29].

The transition from prenatal to postnatal development is marked by dramatic changes in the physiological environment under which the developmental programme unfolds, not least the transition from intra to extra uterine conditions. Under these circumstances the organism faces the challenge of continuing with a normal developmental trajectory under a whole new set of environmental variables.

This adaptation can conceivably demand the widespread remodelling of previously existing regulatory interactions within and between gene networks involved in a wide array of existing and/or emerging cellular and developmental functions. One of the most prominent changes during the perinatal transition is probably the sharp increase in the oxygen concentration and adaptation of the developmental programme to these new conditions could potentially involve the remodelling of existing regulatory interactions involving genes associated to oxygen metabolism. We tested this hypothesis by asking if genes involved in the response to changes in oxygen concentration were significantly overrepresented among genes differentially expressed in the transition from prenatal to postnatal development. To this end we identified differentially expressed genes across the perinatal boundary using the Limma package in R, and searched for enrichment of genes involved in oxygen response. This analysis revealed a significant overrepresentation (FDR < 0.05), among differentially expressed genes, of negative regulation of reactive oxygen species metabolic process (GO:2000378), response to reactive oxygen species (GO:0000302) and reactive oxygen species metabolic process (GO:0072593). A similar result was found using Webgestalt as an alternative tool for GO enrichment analysis [27], also revealing an overrepresentation of genes associated to response to reactive oxygen species (GO:0000302).

We can then test whether this gene expression response to changes in oxygen concentrations during the perinatal transition is in any way associated to an underlying remodelling of previously existing regulatory interactions. We can do this simply by looking at whether oxygen-associated functions were also significantly associated to specific reorganization modules.

A targeted search indeed revealed a significant overrepresentation of GO terms associated to reactivity to oxygen species in specific modules. These include: oxygen metabolic process (GO:0072592, module M10), regulation of response to reactive oxygen species (GO:1901031, module M11), positive regulation of reactive oxygen species metabolic process (GO:2000379, module M3), cellular response to oxygen levels (GO:0071453, module M4), reactive oxygen species metabolic process, and regulation of response to reactive oxygen species (GO:0072593 and GO:1901031, module M9). Similar results were found using Webgestalt (i.e., response to reactive oxygen species GO:0000302, module M3, in addition to significant enrichment of genes involved in response -and positive response- to oxygen levels, GO:0070482 and GO:0036293 respectively in modules M3 and M17).

These results demonstrate that the genetic response to the transition from low to high oxygen concentration during the perinatal boundary is itself associated to an underlying regulatory reorganization of genes involved in oxygen metabolism.

Immediate molecular drivers of the observed regulatory remodelling could in principle include changes in expression of transcription factors specifically targeting individual reorganization modules. Our finding that each reorganization module is targeted by a specific set of transcription factors with very few of them overlapping across modules, provides strong evidence of a potential mechanism driving the reorganization of the global regulatory architecture across the perinatal boundary and further supports to the notion of a re-organization of the underlying regulatory network. If, as suggested by this finding, changes in the pattern of coordinated activity of specific sets of genes is brought about the engagement (or disengagement) of specific sets of transcription factors targeting these gene clusters, we would expect these changes to be mirrored by corresponding changes in the coordinated activation (or down regulation) of numerous transcription factors. This means that we should expect transcription factors to be themselves over represented among clusters of genes undergoing changes in their coordinated activity across the perinatal boundary. Indeed a close inspection of enriched functional categories within the "molecular function" GO term domain reveals a number of reorganization modules with a significant enrichment of transcription factors (thus, for instance, in module M7 we found 24 transcriptional coactivators, 2.4 times more than expected by chance. In module M15 we found 13 genes with transcription regulatory region DNA binding, 2.9 times more than expected by chance, etc.). These results suggest a mechanism whereby changes in the coordinated pattern of activity of numerous transcription factors occurring during the perinatal transition can in turn trigger subsequent changes in the pattern of coordinated activity of specific target modules, all of them involved in a wide array of existing and/or emerging cellular and developmental functions required for the normal transition from prenatal to postnatal brain development.

Additional higher order drivers of the observed regulatory remodelling occurring during the perinatal transition could potentially include epigenetic mechanisms. Among these, DNA methylation is probably the most extensively studied epigenetic modification and has been found involved in many important genomic regulatory processes, including genomic imprinting, X chromosome inactivation, and the regulatory instability of tumour suppressor genes in cancer. In the nervous system, several lines of evidence point to the importance of dynamic epigenetic changes during development [30], with a potentially critical role for DNA methylation in neurodevelopment, as suggested by the dynamic expression of the de novo DNA methyltransferases DNMT3A and DNMT3B during the perinatal period [31]. Along the same lines, a recent analysis of genome-wide patterns of DNA methylation across human foetal brain development uncovered a considerable level of epigenomic plasticity occurring during the immediate prenatal period [32]. While these studies, taken together, highlight the importance of epigenetic reconfiguration events at critical stages in development, further work will be needed to determine their specific contribution to the regulatory reorganization of the transcriptional programme reported in this study.

In the present study, we contrast two models to explain the developmental shift in expression profiles during the transition from prenatal to postnatal development; one based on the assumption of a constant regulatory architecture and the other based on the assumption of a widespread reorganization of the regulatory structure of the developmental transcriptome. It is worth noticing, however, that the two contrasted models should not necessarily be regarded as opposed to each other. Instead they could conceivably be regarded as the two ends of what may be a continuous spectrum. It is also worth noticing that, while the underlying developmental programme is itself as constant a feature as the developmental trajectory itself, given the complexity of the developmental process, an entirely static regulatory structure is not necessarily expected to start with. However the fact remains that instead of a series of gradual shifts in the regulatory structure of the developmental programme, we find two well defined regulatory architectures operating at either side of the perinatal boundary. In other words a remarkable regulatory stability is indeed observed across time and regions during prenatal development, followed by a major regulatory shift during the prenatal-postnatal transition leading to a second stable regulatory architecture during postnatal development.

## Conclusions

In sum, we conclude that, during brain development, the pronounced changes in the genome wide expression profile observed in the perinatal boundary are the result of a regulatory reorganization of the developmental programme occurring at birth and reflecting the reassembly of new functional and regulatory associations required for the normal transition from prenatal to postnatal nervous system development.

## Methods

### Expression data

RNAseq RPKM-normalized expression data summarized to genes was obtained from NIMH Transcriptional Atlas of Human Brain Development (http://www.brainspan.org/). We selected 112 samples corresponding to eight brain structures for which there was available data across 14 early stages. This resulted in the following cortical regions: Dorsolateral prefrontal cortex (DFC), Posteroinferior (ventral) parietal cortex (IPC), Anterior (rostral) cingulate (medial prefrontal) cortex (MFC), Orbital frontal cortex (OFC), Primary somatosensory cortex (S1C), Posterior (caudal) superior temporal cortex (STC), Primary visual cortex (V1C) and Ventrolateral prefrontal cortex (VFC). Seven of the fourteen different developmental stages correspond to post-conception weeks 12, 13, 16, 17, 21, 24 and 37. The other seven postnatal time points are 4 months after birth followed by 1, 2, 3, 8, 11 and 13 years of age. We selected only protein coding genes according to the Ensembl version 77 annotations and removed from the analysis all genes displaying zero variance across samples resulting in a total of 18526 genes.

### Expression profile clustering analysis

To quantify similarity of expression profiles across brain structures and between two developmental stages (prenatal and postnatal), we obtained the Pearson correlation coefficient (R) between the normalized average expression values per gene per structure of all possible pairs of expression profiles. We defined distance between any two expression profiles as 1-R and performed average linked hierarchical clustering analysis.

### Coexpression structure clustering analysis

To measure the degree of similarity in the coexpression structure of the same set of brain regions at both prenatal and postnatal stages, we compared the coexpression matrices obtained for all regions at both prenatal and postnatal windows. More specifically, for each cortical region, we obtained the coexpression matrix (defined as the Pearson correlation matrix between all possible pairs of genes) across all seven prenatal time points. We repeated the same procedure for all postnatal time points resulting in a total of 16 global coexpression matrices (eight prenatal and eight postnatal brain regions). We defined similarity between any two coexpression matrices as 1-R, where R is the Pearson correlation coefficient resulting from directly comparing any two coexpression matrices. The resulting similarity indexes were used to perform a hierarchical clustering analysis.

### Differential coexpression analysis

To quantify changes in the global pattern of coexpression in the perinatal boundary, we performed differential coexpression analysis as described by Tesson [26] based on a Weighted Gene Correlation Network Analysis (WGCNA) approach. Briefly; we calculated correlation coefficients for all possible gene pairs separately for the prenatal and postnatal period, obtaining one global correlation matrix for each stage. Then we computed the adjacency difference matrix using the soft threshold parameter β = 6 (in order to achieve a scale–free degree distribution with fitting index R^2^ > 0.8). Next, hierarchical clustering was performed based on the Topological Overlap of the difference matrix. Finally, the *dynamic tree cut* function (implemented in R) was used in order to identify gene modules (minimum cluster size of 100 genes deep split = TRUE). Modules where merged when the module’s eigengenes correlation was higher than *r* = 0.9.

### Gene Ontology and transcription factor target enrichment analysis

We downloaded gene ontology biological process (GO) annotations from Ensembl version 77 (http://www.ensembl.org/index.html), and selected only those GO terms containing at least 150 genes for which expression data was available. Enrichment analysis for each of the modules detected through differential coexpression analysis was carried out as described elsewhere [33]. Briefly; statistically significant overrepresentation of GO terms was assessed based on a Z-score test. Mean and standard deviation for the expected number of genes annotated to each GO term per module were estimated based on 10,000 equally-sized random samples drawn from the background gene population. P values, where adjusted for multiple testing using Benjamini-Hochberg correction and GO enrichments were deemed significant when FDR < 0.05 and the difference between observed and expected genes was larger than one. Enrichment analyses based on an alternative (hypergeometric) test were carried out using Webgestalt [27].

Transcription factor binding site enrichment analysis was carried out using the transcription factor target analysis toolkit from Webgestalt [27].

To represent the distribution and overlap of transcription factor targets across modules, a Venn and Euler diagram was generated using the *venneuler* function supported in R, where areas are proportional to the number of significantly enriched transcription factor targets per module and overlap areas are proportional to the number of overlapping transcription factors between modules.

#### Differential expression analysis

Differentially expressed genes between prenatal and postnatal samples were identified on RPKM expression values using the Limma package supported by R. The resulting set of differentially expressed genes was then used to specifically assess enrichment of the following oxygen metabolism-associated GO IDs: GO:2000376, GO:2000378, GO:0072592, GO:0000302, GO:0034614, GO:0072593, GO:2000377, GO:2000379, GO:0015671, GO:0071453, GO:0052567, GO:0032364, GO:0001315, GO:0000305, GO:1901031, GO:0036294.

### Programming and statistical software

All large scale calculations, numerical simulations and statistical analyses were carried out in R. The Differential coexpression analysis script was obtained from Tesson et al (2010) [26] already implemented in R. Network visualization was implemented in Gephi using the inbuilt Fruchterman-Reingold layout.

## Additional files

Additional file 1: Expression profile similarity heatmap corresponding to the regional profiles examined in figure 1 of the main text. Each cortical region and stage (either prenatal or postnatal) is indicated in left and bottom labels. Pearson correlation coefficient between each possible pair of expression profiles are indicated in the heatmap matrix. Blue colour denotes positive correlation, yellow correlations equal to zero and red negative correlations. (PDF 32 kb) Additional file 2: Expression profile Heatmap showing the average expression for over 18,000 genes for each examined cortical structure and each developmental stage (prenatal or postnatal) with the accompanying dendrogram showing the similarity between these expression profiles. The gene expression has been normalized per gene, green and red denotes high and low gene respectively. Each cortical region and stage (either prenatal or postnatal) is indicated at the bottom. Inset shows Z-score colour key histogram. (PDF 2586 kb) Additional file 3: Heatmap showing similarity between co-expression profiles. We obtained the coexpression matrices of each brain region for prenatal and postnatal stages separately, resulting in a total of 16 different coexpression matrices: one for each of the eight brain regions at either prenatal or postnatal stages. Pearson correlation coefficient between each possible pair of co-expression matrices are indicated in the heatmap. Correlation colour scale is indicated: Blue colour denotes positive correlation, yellow correlations equal to zero and red negative correlations. Each cortical region and stage (either prenatal or postnatal) is indicated in left and bottom labels. (PDF 32 kb) Additional file 4: Gene ontology enrichment analysis per module. Only gene ontology terms annotated to at least 150 genes are included. Statistically significant overrepresentation of GO terms was assessed based on a Z-score test. Mean and standard deviation for the expected number of genes annotated to each GO term per module were estimated based on 10,000 equally-sized random samples drawn from the background gene population. P values, where adjusted for multiple testing using Benjamini-Hochberg correction. Separate tabs are included in this file for each separate module. (XLSX 41 kb) Additional file 5: Gene ontology enrichment analysis per module obtained using Webgestalt [27]. Statistically significant overrepresentation of GO terms was assessed based on the tool’s standard hypergeometric test. Only biological process terms were considered. Separate tabs are included in this file for each separate module. (XLSX 235 kb) Additional file 6: Transcription factor binding site enrichment analysis per module obtained using Webgestalt [27]. Statistically significant overrepresentation of transcription factor binding motifs terms was assessed based on the tool’s standard hypergeometric test. Separate tabs are included in this file for each separate module. (XLSX 27 kb)

## Abbreviations

DFC: Dorsolateral prefrontal cortex
IPC: Posteroinferior (ventral) parietal cortex
MFC: Anterior (rostral) cingulate (medial prefrontal) cortex
OFC: Orbital frontal cortex (OFC)
S1C: Primary somatosensory cortex (area S1, areas 3, 1, 2)
STC: Posterior (caudal) superior temporal cortex (area TAc)
V1C: Primary visual cortex (striate cortex, area V1/17)
VFC: Ventrolateral prefrontal cortex
