# Supplementary figures and images for: Modular reorganization of the global network of gene regulatory interactions during perinatal human brain development
Source: BMC Dev Biol. 2016 May 12;16:13. doi: 10.1186/s12861-016-0111-3 (PMC4866393; doi:10.1186/s12861-016-0111-3)

Expression profile similarity matrix

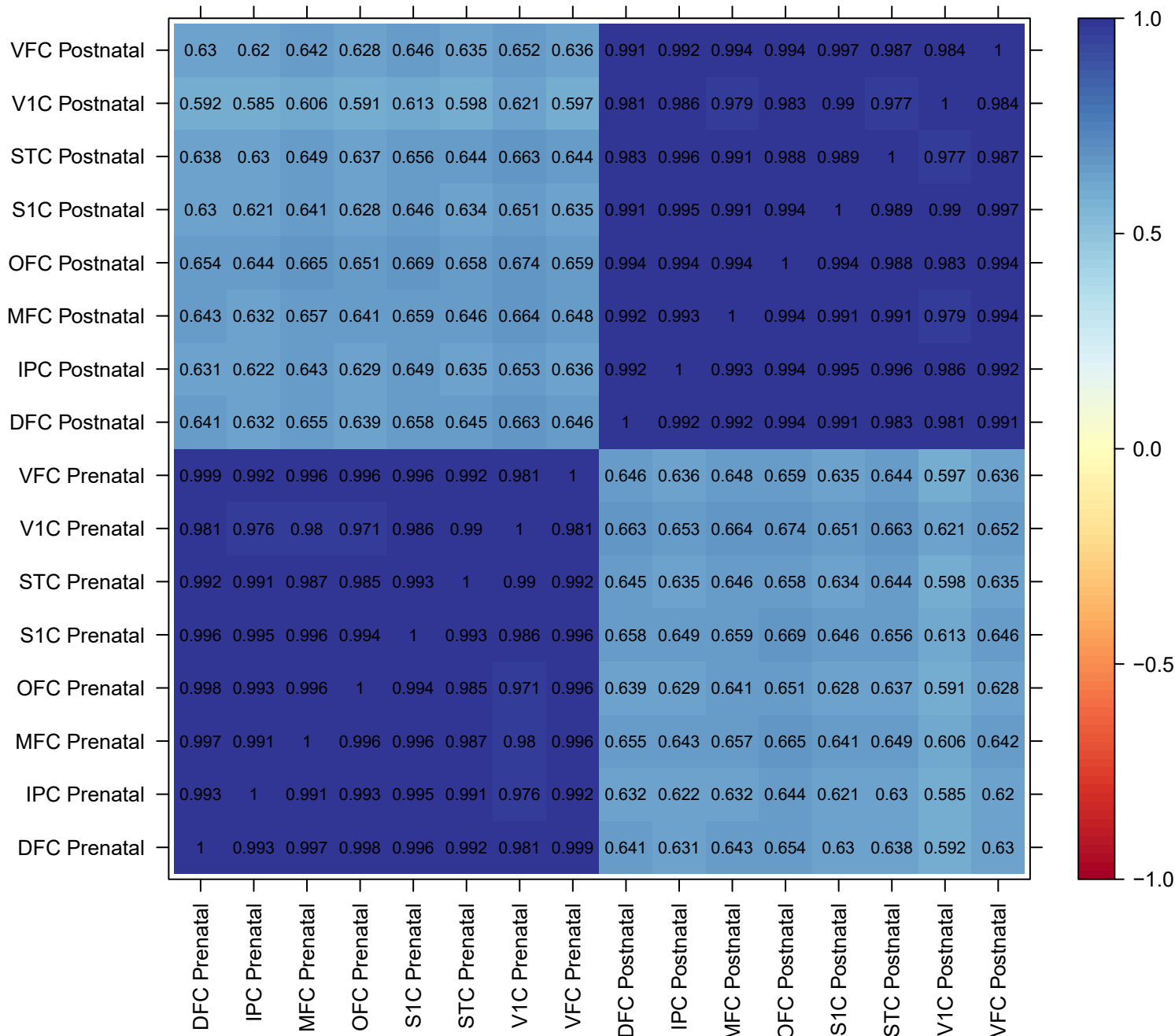

Supplementary figure 1

Supplement: Additional file 1: — Expression profile similarity heatmap corresponding to the regional profiles examined in figure 1 of the main text. Each cortical region and stage (either prenatal or postnatal) is indicated in left and bottom labels. Pearson correlation coefficient between each possible pair of expression profiles are indicated in the heatmap matrix. Blue colour denotes positive correlation, yellow correlations equal to zero and red negative correlations. (PDF 32 kb) [file 12861_2016_111_MOESM1_ESM.pdf]

# Expression profile heatmap

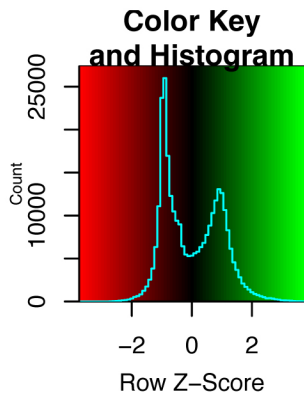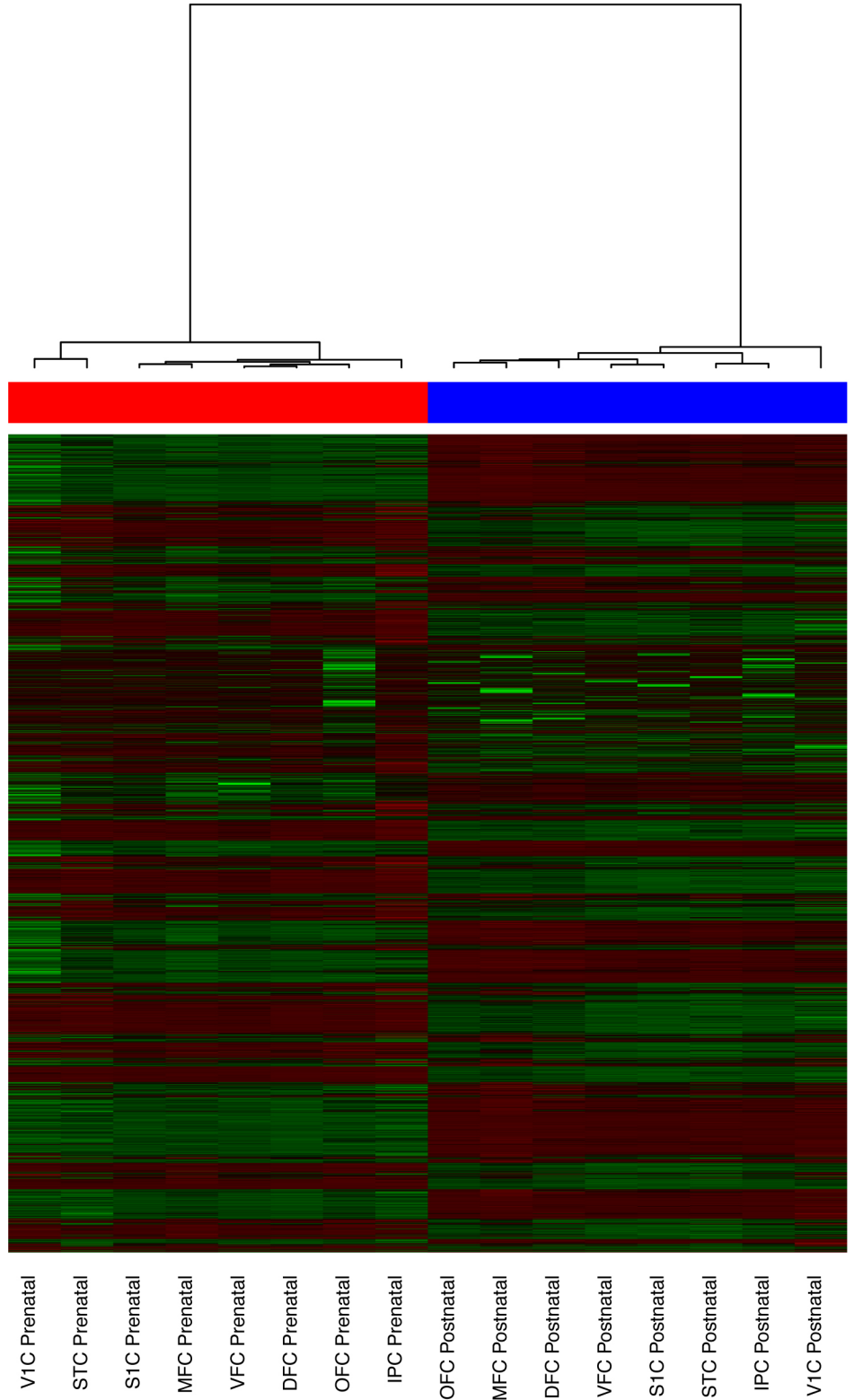

Supplement: Additional file 2: — Expression profile Heatmap showing the average expression for over 18,000 genes for each examined cortical structure and each developmental stage (prenatal or postnatal) with the accompanying dendrogram showing the similarity between these expression profiles. The gene expression has been normalized per gene, green and red denotes high and low gene respectively. Each cortical region and stage (either prenatal or postnatal) is indicated at the bottom. Inset shows Z-score colour key histogram. (PDF 2586 kb) [file 12861_2016_111_MOESM2_ESM.pdf]

# Coexpression profile similarity matrix

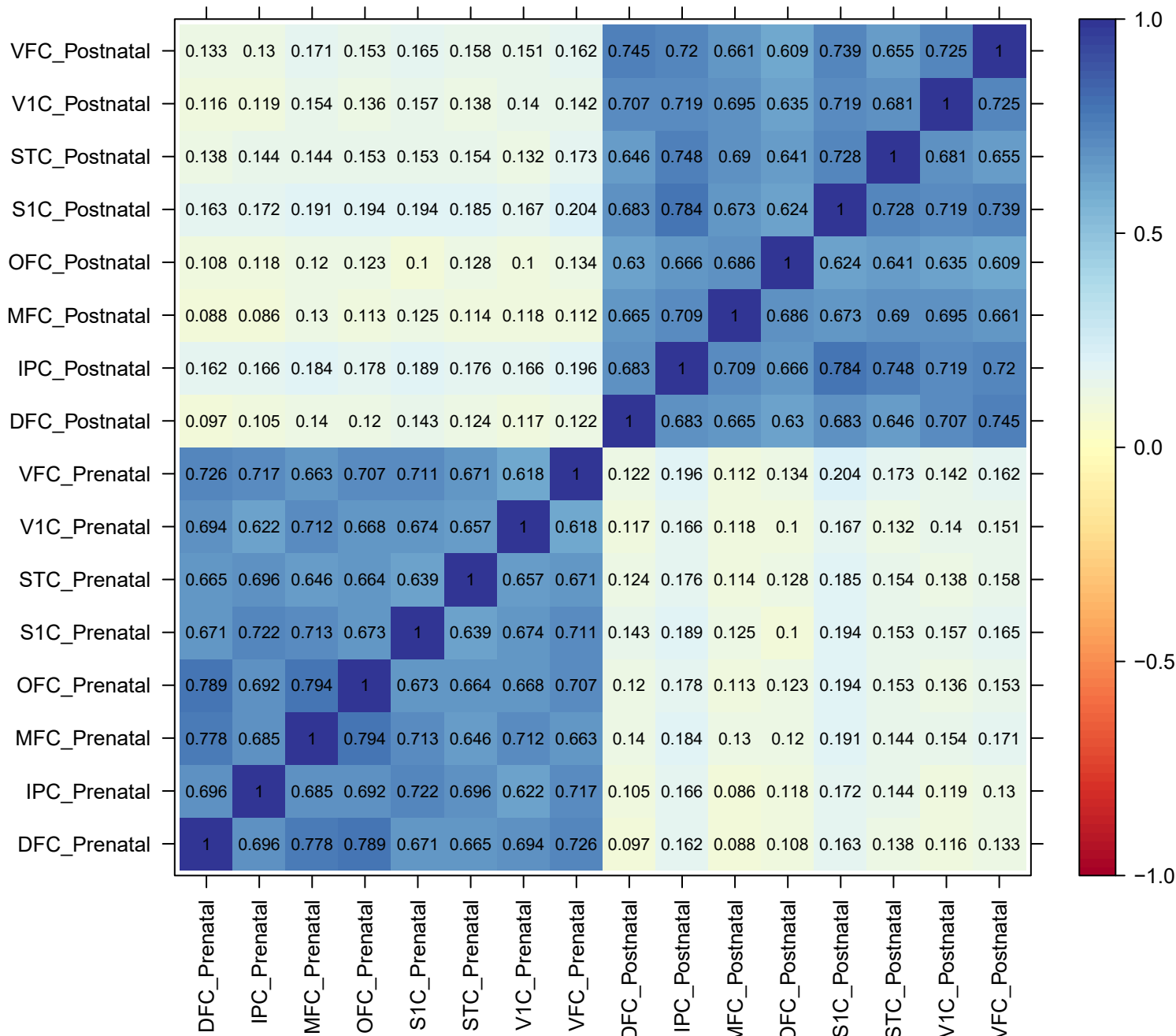

Supplement: Additional file 3: — Heatmap showing similarity between co-expression profiles. We obtained the coexpression matrices of each brain region for prenatal and postnatal stages separately, resulting in a total of 16 different coexpression matrices: one for each of the eight brain regions at either prenatal or postnatal stages. Pearson correlation coefficient between each possible pair of co-expression matrices are indicated in the heatmap. Correlation colour scale is indicated: Blue colour denotes positive correlation, yellow correlations equal to zero and red negative correlations. Each cortical region and stage (either prenatal or postnatal) is indicated in left and bottom labels. (PDF 32 kb) [file 12861_2016_111_MOESM3_ESM.pdf]
